# Choosing source of microorganisms and processing technology for next generation beet bioinoculant

**DOI:** 10.1038/s41598-021-82436-5

**Published:** 2021-02-02

**Authors:** Sonia Szymańska, Marcin Sikora, Katarzyna Hrynkiewicz, Jarosław Tyburski, Andrzej Tretyn, Marcin Gołębiewski

**Affiliations:** 1 Department of Microbiology, Faculty of Biological and Veterinary Sciences, Nicolaus Copernicus University (NCU), Lwowska 1, 87-100 Toruń, Poland; 2 Center for Modern Interdisciplinary Technologies, Nicolaus Copernicus University (NCU), Toruń, Poland; 3 Chair of Plant Physiology and Biotechnology, Faculty of Biological and Veterinary Sciences, Nicolaus Copernicus University (NCU), Lwowska 1, 87-100 Toruń, Poland

**Keywords:** Plant biotechnology, Microbiome, Microbial ecology

## Abstract

The increase of human population and associated increasing demand for agricultural products lead to soil over-exploitation. Biofertilizers based on lyophilized plant material containing living plant growth-promoting microorganisms (PGPM) could be an alternative to conventional fertilizers that fits into sustainable agricultural technologies ideas. We aimed to: (1) assess the diversity of endophytic bacteria in sugar and sea beet roots and (2) determine the influence of osmoprotectants (trehalose and ectoine) addition during lyophilization on bacterial density, viability and salt tolerance. Microbiome diversity was assessed based on 16S rRNA amplicons sequencing, bacterial density and salt tolerance was evaluated in cultures, while bacterial viability was calculated by using fluorescence microscopy and flow cytometry. Here we show that plant genotype shapes its endophytic microbiome diversity and determines rhizosphere soil properties. Sea beet endophytic microbiome, consisting of genera characteristic for extreme environments, is more diverse and salt resistant than its crop relative. Supplementing osmoprotectants during root tissue lyophilization exerts a positive effect on bacterial community salt stress tolerance, viability and density. Trehalose improves the above-mentioned parameters more effectively than ectoine, moreover its use is economically advantageous, thus it may be used to formulate improved biofertilizers.

## Introduction

Conventional agriculture practices negatively affect environment, e.g. by decreasing microbial diversity, soil quality, water supply and plant productivity^1,2^. Wide adoption of sustainable agricultural technologies, e.g. biofertilizers, may significantly decrease the use of chemical fertilizers, reducing negative consequences of agriculture on the environment^2,3^.

Biofertilizers are based on living plant growth-promoting microorganisms (arbuscular mycorrhizal fungi—AMF, plant growth-promoting rhizobacteria—PGPR, nitrogen fixing bacteria—NFB) and are key players in sustainable agriculture^4^. They can promote plant growth in several different ways (e.g. increasing availability of nutrients, synthesizing phytohormones or siderophores, fixing nitrogen), especially under unfavorable environmental conditions (e.g. drought or salinity)^5–7^. Most of commercially available biofertilizers are based on combination of two or more microbial beneficial strains, which is called ‘consortium’^4^. Compared to single strains, consortia display increased spectrum of beneficial effect of inoculum on plants. However, criteria of strain selection are the crucial factor influencing the inoculum success and should be considered not only based on plant genotype compatibility but also environmental factors.

The methods of biofertilizers production, storage and application are diverse. Inoculation techniques are based on microorganisms application in liquid (sprays and drenches) or solid form (lyophilizates delivered to soil/growth substrate). The most important problem in the preparation and storage technology of biofertilizers is maintaining high viability of microorganisms^8^. Lyophilization is well known and widely used technique extending microbial cell viability^9^. To alleviate negative effect of low temperature and desiccation on microorganisms in this technology, several different stabilizers can be used e.g. nonreducing disaccharides, glicerolglycerol or skim milk^10^.

Trehalose (α-d-glucopyranosyl-(1 → 1)-α-d-glucopyranoside) is a disaccharide present in almost all prokaryotic and eukaryotic organisms and exhibits high efficiency in protection of cells against low temperature, drying and osmotic stress^10–12^. Ectoine (1,4,5,6-tetrahydro-2-methyl-4-pyrimidinecarboxylic acid) is synthesized mostly by halotolerant and halophilic microorganisms and responsible for regulation of osmotic pressure in microbial cells, increasing their tolerance to osmotic stress (salinity)^13–16^. Application of trehalose and ectoine in the process of lyophilization of endophytic microbiomes was tested in our work for the first time.

Biofertilizer efficiency analyzed under laboratory conditions may not correspond to results obtained under field conditions^2,17^. This effect may be due to adverse effect of environmental conditions or autochthonic microorganisms on gene expression in microbial cells^2^ or low competitiveness of microorganisms used as biofertilizers, i.e. they may be outgrown by the autochthonic ones. This is why “plant microbiome” was proposed as the new generation of inoculants^18^. Inoculation of crops with microbiome and organic matter present in lyophilized plant roots seems to be a better solution to enrich microbial biodiversity of soil and crops with new endophytes.

Endophytes are bacteria and fungi that colonize the internal plants tissues without causing pathogenic symptoms^19^ and can directly (nitrogen fixation, phosphate solubilization, siderophore and phytohormone synthesis) and/or indirectly (biocontrol agents) promote plant growth and development, e.g. in crops^20^.

Recent data show that more than 7% of global land surface and 70% of all irrigated agricultural soils worldwide is affected by salinity^21^, and the problem is exacerbated by inorganic fertilization as well as by climate changes. Moreover, halophytes thriving in naturally saline environments are reservoirs of endophytes possessing high tolerance to salt stress^22,23^ that may be useful in alleviation of salt stress in crops. Application of halotolerant microbes in sustainable agriculture e.g. in the increasing salinity tolerance of non-halophytic crops, is well known and was extensively studied^6,24–27^.

Cultivated beets are one of the few crops whose direct ancestor (sea beet, *Beta vulgaris* ssp. *maritima*) still grows in the wild. This feature enables comparison of traits in plants that are very close genetically (ca. 0.5% difference^28^), but whose ecology differs considerably. Moreover, as sea beet is as a halophyte growing in nature^29^, it seems to be a good candidate for a source of microorganisms that could be useful for crop beets improvement. We analyzed both rhizosphere soil and plant roots to determine if the plant influence on the former is strong enough to make it the source of microbes for bioinoculant formulation.

The goal of our study was twofold: (i) to characterize rhizosphere and root microbiomes of cultivated and wild beet and choose the source of microorganisms for prospective bioinoculant formulation for growing beet in saline soils and (ii) to check if addition of osmoprotectants during lyophilization changes root bacterial community structure as well as microbiome salinity tolerance and viability. Specifically, we formulated two general hypotheses: (i) roots of different beet genotypes would filter rhizosphere microbes specifically and therefore they would be better source of microbes for bioinoculation than rhizosphere soils, (ii) addition of osmoprotectants would allow formulation of a better inoculant because of increased bacterial viability and microbiome salinity tolerance.

## Results

### Rhizosphere as a source of microbes in endosphere

#### Rhizosphere soil physicochemical parameters are different for sugar and sea beet

Majority of tested parameters was higher in sugar beet soil, but only in cases of CaCO_3_ and Na^+^ the difference was statistically significant. On the other hand, OC, P, Ca^2+^, Mg^2+^ and N_t_ were higher in sea beet soil and for the latter the difference was significant (Table 1). To check if these changed parameters influenced bacterial communities in rhizosphere soils, we sequenced 16S rRNA amplicon libraries.

**Table 1:** Physico-chemical rhizosphere soil parameters (mean and standard deviation) obtained after three months of cultivation of sugar- and sea beet.

Parameter\plant genotype	*cv. ’Huzar’*	*B. maritima*
OC (%)	4.97 (1,646)	5.66 (1,210)
N_t_ (%)	0.28 (0.032)	0.34 (0.025) [↑]
CaCO_3_ (%)	1.88 (0.178) [↑]	1.6 (0.107)
P_citr._ [mg/kg]	1183,08 (116,312)	1253,29 (48.876)
pH	7.1 (0.077)	7.0 (0.074)
EC 1:5 [µS·cm^−1^]	176,93 (57,826)	142,28 (23,973)
Na^+^ [mg·dm^−3^]	16,80 (7,652) [↑]	7,35 (1,885)
K^+^ [mg·dm^−3^]	2,95 (0,644)	2,55 (1,111)
Ca^2+^ [mg·dm^−3^]	9,63 (2,291)	12,93 (2,916)
Mg^2+^ [mg·dm^−3^]	1,28 (0,306)	1,52 (0,223)
Cl^−^ [mg·dm^−3^]	46,02 (15,494)	38,94 (2,890)
SO_4y_^2−^ [mg·dm^−3^]	39,35 (5,211)	38,58 (7,998)
HCO_3_^−^ [mg·dm^−3^]	94,55 (14,306)	88,45 (29,063)

[↑] significantly higher level based on Newman-Keuls test of rhizosphere soil parameter observed between the plant species.

#### Bacterial diversity in sugar beet roots is lower than in its wild ancestor

**Figure 1:**
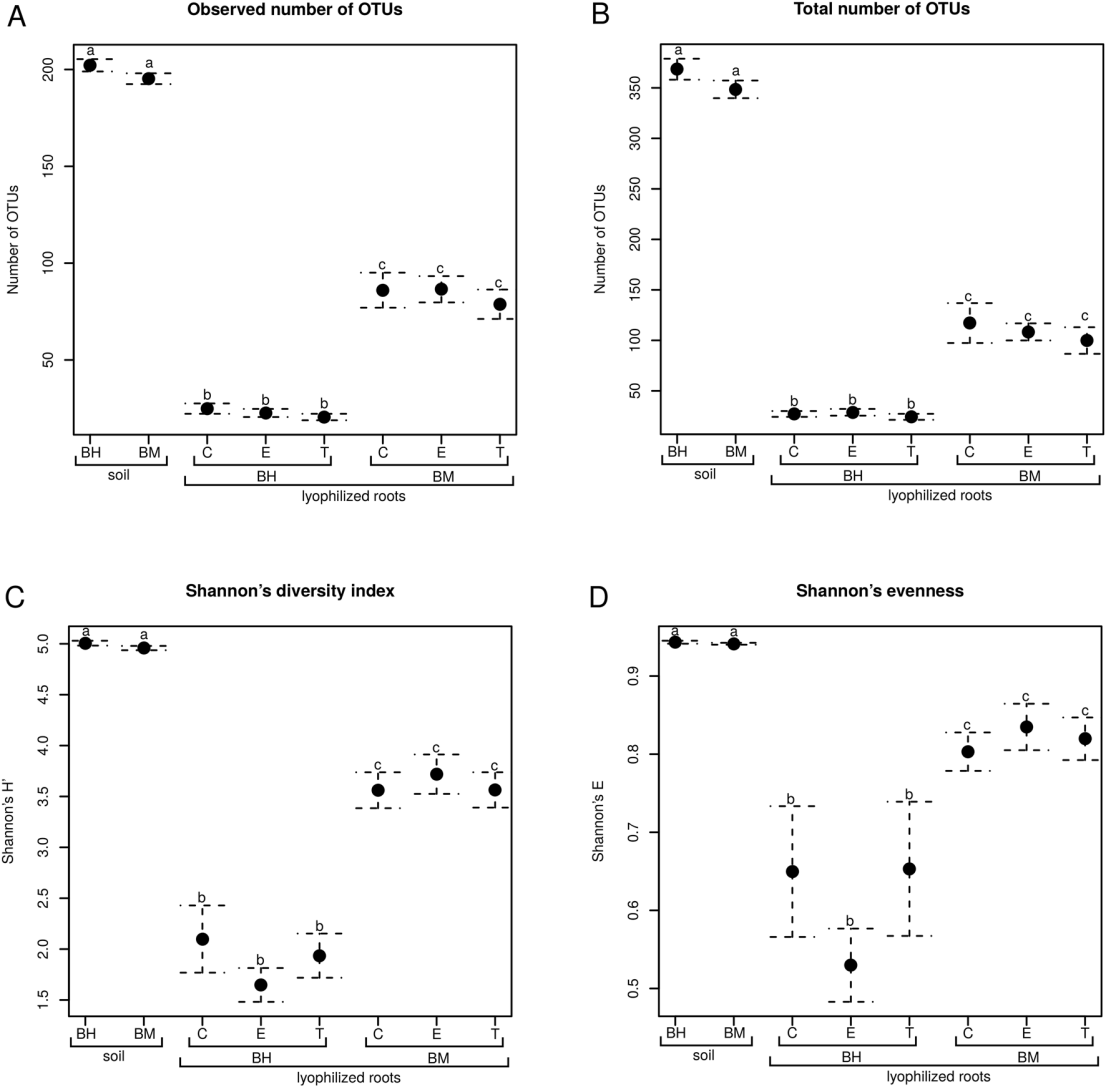
Species richness, evenness and diversity of bacterial communities in rhizosphere soils of sugar beet (Bh) and sea beet (Bm) and lyophilized roots of these plants untreated (C), and treated with ectoine (E) or trehalose (T). Means (n = 8–32) are presented, whiskers show standard error of the mean (SEM), and significant differences (ANOVA, p < 0.05) are denoted with different letters. Observed number of OTUs (**A**), estimated total number of OTUs (Chao1 index, **B**), Shannon's diversity index (H', **C**), Shannon's evenness (**D**).

Bacterial diversity, evenness and species richness were the highest in 16S rRNA libraries coming from rhizosphere soil, regardless of plant genotype. Lyophilized sea beet roots harbored more diverse community than sugar beet (Fig. 1). The number of OTUs was ca. three times higher in the wild beet than in the crop (Fig. 1A,B), while the diversity was around 1.5 times higher (Fig. 1C), and evenness was ca. 1.3 times greater (Fig. 1D).

#### Both endophytic and rhizosphere soil bacterial community is dominated by Proteobacteria

**Figure 2:**
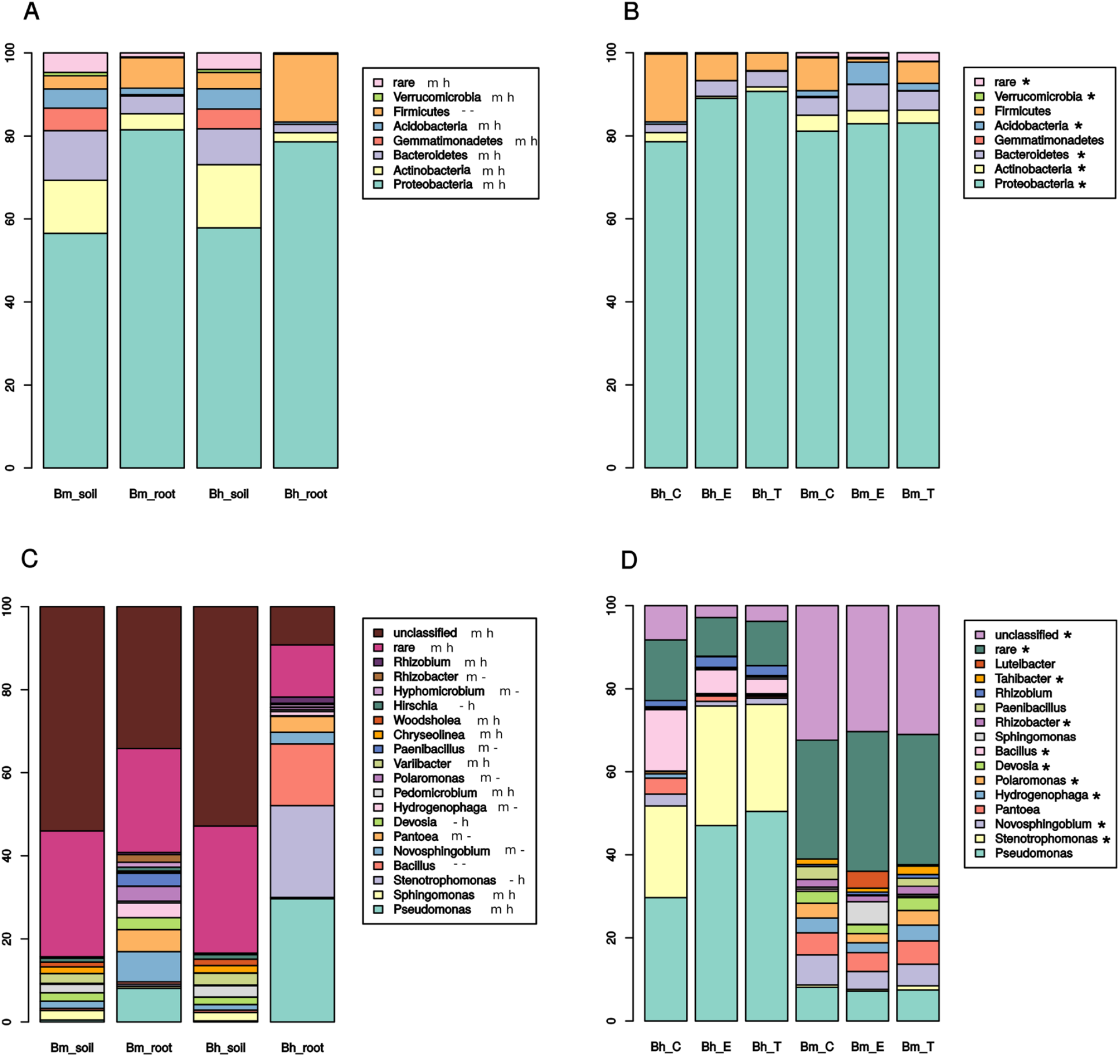
Taxonomic composition of bacteria communities in rhizosphere soils of sugar beet (Bh) and sea beet (Bm) (**A**, **C**) and lyophilized roots of these plants (**B**, **D**) untreated (Bh_C and Bm_C) and treated with ectoine (Bh_E, Bm_E) or trehalose (Bh_T, Bm_T) at the phylum (**A**, **B**) and genus (**C**, **D**) levels. Means (n = 8–32) are presented, and significant differences between genotypes are marked either with m and h letters (panels **A** and **C**, significant differences between rhizosphere and endosphere in sea (m) or sugar (h) beet) or with asterisks (panels **B** and **D**, significant differences between genotypes, no differences due to osmolytes were found).

There were no significant differences in taxonomic composition of rhizosphere soil bacterial communities of sugar- and sea beet at the level of phylum (Fig. 2A). At the level of genus, three taxa were differentially represented, all of them belonging to Alphaproteobacteria: two Rhizobiales-belonging genera, *Pedomicrobium* and an unknown genus of JG34.KF.361 family as well as *Woodsholea* (Caulobacteraceae) were more abundant in the crop (Fig. 2C). Differences in lyophilized roots communities were more pronounced, although still there were no taxa significantly differentially represented between osmolyte treatments. At the level of phyla Proteobacteria-derived reads were more abundant in libraries from sugar beet lyophilized roots, while Actinobacteria, Bacteroidetes, Acidobacteria, Verrucomicrobia and rare phyla were more abundant in its wild ancestor (Fig. 2B). Among genera significant differences were observed for *Stenotrophomonas* and *Bacillus* that were more abundant in the crop and for proteobacterial genera *Novosphingobium*, *Devosia* (Alphaproteobacteria), *Hydrogenophaga*, *Polaromonas* (Betaproteobacteria), *Rhizobacter* and *Tahibacter* (Gammaproteobacteria) as well as for rare and unclassified genera being more abundant in sea beet (Fig. 2D).

At the level of phyla , regardless of plant genotype, there were significant differences between soil and roots in all taxa but Firmicutes. Proteobacteria and Firmicutes were more abundant in roots than in soil, while abundance of the remaining phyla was lower *in planta*, and Gemmatimonadetes as well as Verrucomicrobia were absent from roots. At the level of genus, regardless of genotype, *Pseudomonas* and *Rhizobium* were significantly more abundant in roots than in soil, while *Sphingomonas*, *Pedomicrobium*, rare and unclassified bacteria were less frequent in roots than in rhizosphere. *Novosphingobium*, *Pantoea*, *Hydrogenophaga*, *Polaromonas*, *Paenibacillus*, *Hyphomicrobium* and *Rhizobacter* were significantly more abundant in wild beet roots than in soil, while *Stenotrophomonas* was the only genus that was more frequent in sugar beet roots than in soil. *Variibacter*, *Chryseolinea*, and *Woodsholea* were less abundant in wild beet roots than in soil, while *Devosia* and *Hirschia* were less frequent in sugar beet than in soil.

### Effect of osmolytes on diversity, viability, and tolerance to salinity of bacterial communities in lyophilized beet roots

#### Bacterial cell density in lyophilized roots depends on host genotype but not on osmolyte

**Figure 3:**
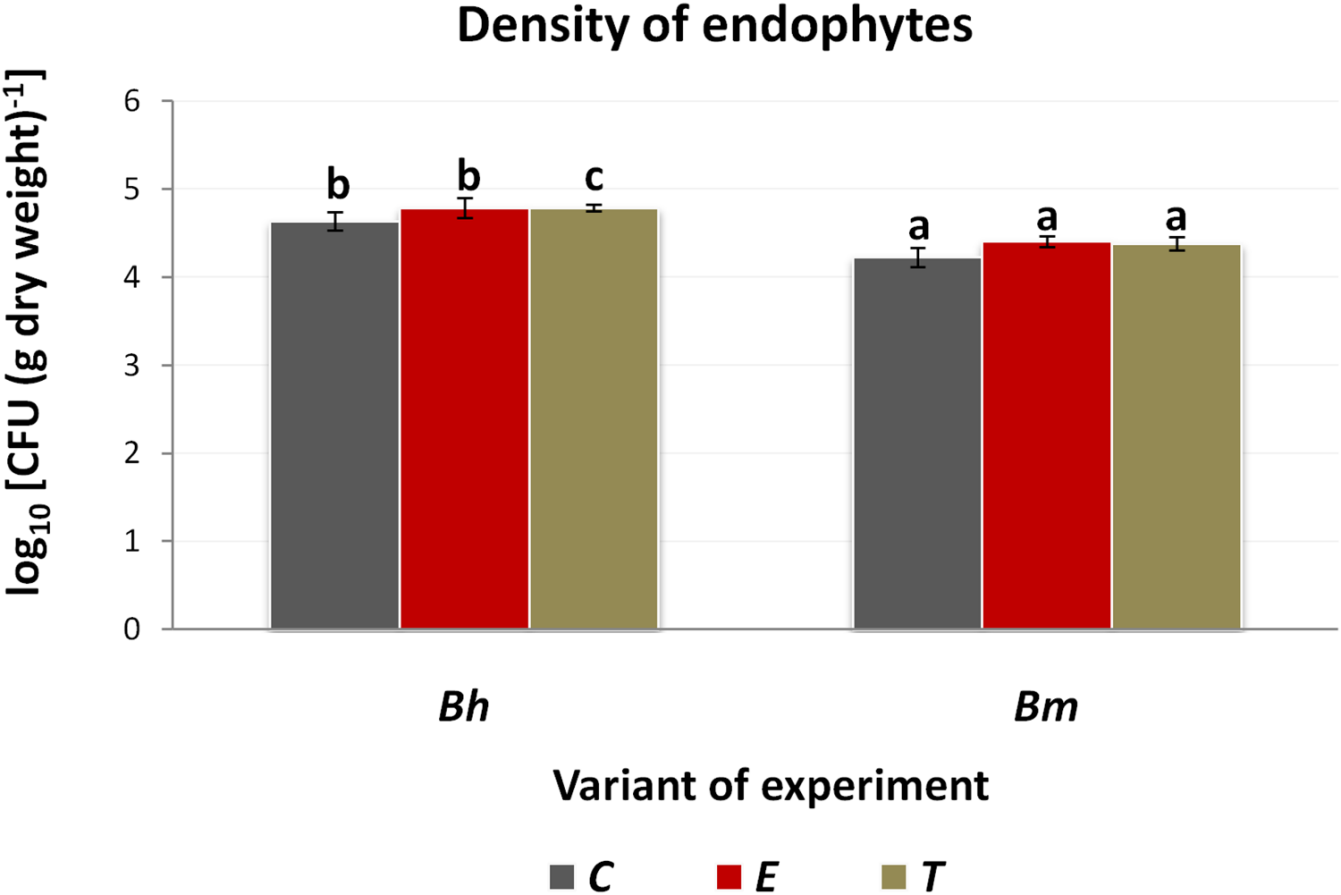
Density of endophytic bacteria (expressed as log_10_ CFU per g of dry weight) isolated from lyophilized sugar- and sea beet roots. Means (n = 3) ± standard deviation are presented. Significant differences between variants (ANOVA, p < 0.05, with Tukey's HSD; C–control untreated with any osmolyte, ectoine (E) or trehalose (T) treated) were marked with different letters.

In total, 72 bacterial strains were isolated and identified, 35 coming from sugar beet and 37 from sea beet. Proteobacteria were the most frequent phylum in fresh roots of both sugar and sea beet, followed by Actinobacteria in the crop and Firmicutes in the wild plant. *Pseudomonas* and *Sphingomonas* were characteristic for fresh roots of sugar beet, while *Bosea* and *Sphingopyxis* were found exclusively in sea beet roots before lyophilization (Table 2). Density of culturable root endophytic bacteria was higher in sugar beet lyophilizates than in sea beet (ANOVA, p < 0.05, Fig. 3), regardless of the osmolytes addition. We observed no influence of osmolytes on sea beet endophytes density, while trehalose increased slightly, but significantly (ANOVA, p < 0.05) the density in sugar beet samples (Fig. 3).

**Table 2:** Identification of cultivable endophytic bacteria associated with roots of sugar- and sea beet before and after lyophilization without addition of any osmolyte (C) or supplemented either with ectoine (E) or trehalose (T).

After lyophilization	Genotype	
Treatment	*cv. ‘Huzar’*	*B. maritima*
*C*		
1	*Gordonia* sp. BH1CTR8 (A)	*Bacillus* sp. BM1CTR1 (F)
2	*Bacillus* sp. BH1CTR2 (F)	*Bacillus* sp. BM1CTR10 (F)
3	*Bacillus* sp. BH1CTR5 (F)	*Bacillus* sp. BM1CTR9 (F)
4	*Bacillus* sp. BH3CTR4 (F)	*Bacillus* sp. BM3CTR10 (F)
5	*Paenibacillus* sp. BH3CTR10 (F)	*Bacillus* sp. BM3CTR4 (F)
6	*Acinetobacter* sp. BH2CTR5 (P)	*Psychrobacillus* sp. BM3CTR11 (F)
7	*Pantoea* sp. BH4CTR1 (P)	
8	*Pseudoxanthomonas* sp. BH3CTR6 (P)	
9	*Pseudoxanthomonas* sp. BH3CTR9 (P)	
10	*Shinella* sp. BH1CTR1 (P)	
*E*		
1	*Bacillus* sp. BH1EKT5 (F)	*Bacillus* sp. BM1EKT11 (F)
2	*Bacillus* sp. BH1EKT9 (F)	*Bacillus* sp. BM1EKT2 (F)
3	*Bacillus sp.* BH4EKT3 (F)	*Bacillus* sp. BM2EKT5 (F)
4	*Bacillus* sp. BH4EKT5 (F)	*Bacillus* sp. BM4EKT1 (F)
5	*Pseudoxanthomonas* sp. BH1EKT3 (P)	*Bacillus* sp. BM4EKT10 (F)
6	*Pseudoxanthomonas* sp. BH5EKT5 (P)	*Shinella* sp. BM1EKT6 (P)
7	*Sphingobium* sp. BH1EKT10 (P)	*Stenotrophomonas* sp. BM4EKT2 (P)
8	*Sphingobium* sp. BH2EKT1 (P)	*Stenotrophomonas* sp. BM4EKT3 (P)
9		*Stenotrophomonas* sp. BM4EKT5 (P)
10		*Stenotrophomonas* sp. BM4EKT8 (P)
*T*		
1	*Bacillus* sp. BH3TRE4 (F)	*Nocardiopsis* sp. BM4TRE1
2	*Pantoea* sp. BH4TRE2 (P)	*Bacillus* sp. BM1TRE9 (F)
3	*Pantoea* sp. BH4TRE3 (P)	*Bacillus* sp. BM3TRE11 (F)
4	*Pseudomonas* sp. BH4TRE5 (P)	*Bacillus* sp. BM3TRE8 (F)
5	*Pseudomonas* sp. BH4TRE1 (P)	*Bacillus* sp. BM4TRE10 (F)
6	*Shinella* sp. BH2TRE2 (P)	*Bacillus* sp. BM4TRE4 (F)
7	*Shinella* sp. BH2TRE3 (P)	*Pseudomonas* sp. BM1TRE2 (P)
8		*Pseudomonas* sp. BM3TRE2 (P)
9		*Pseudoxanthomonas* sp. BM1TRE1 (P)
10		*Shinella* sp. BM3TRE3 (P)

#### Sea beet endophytes are more salt tolerant than sugar beet ones

**Figure 4:**
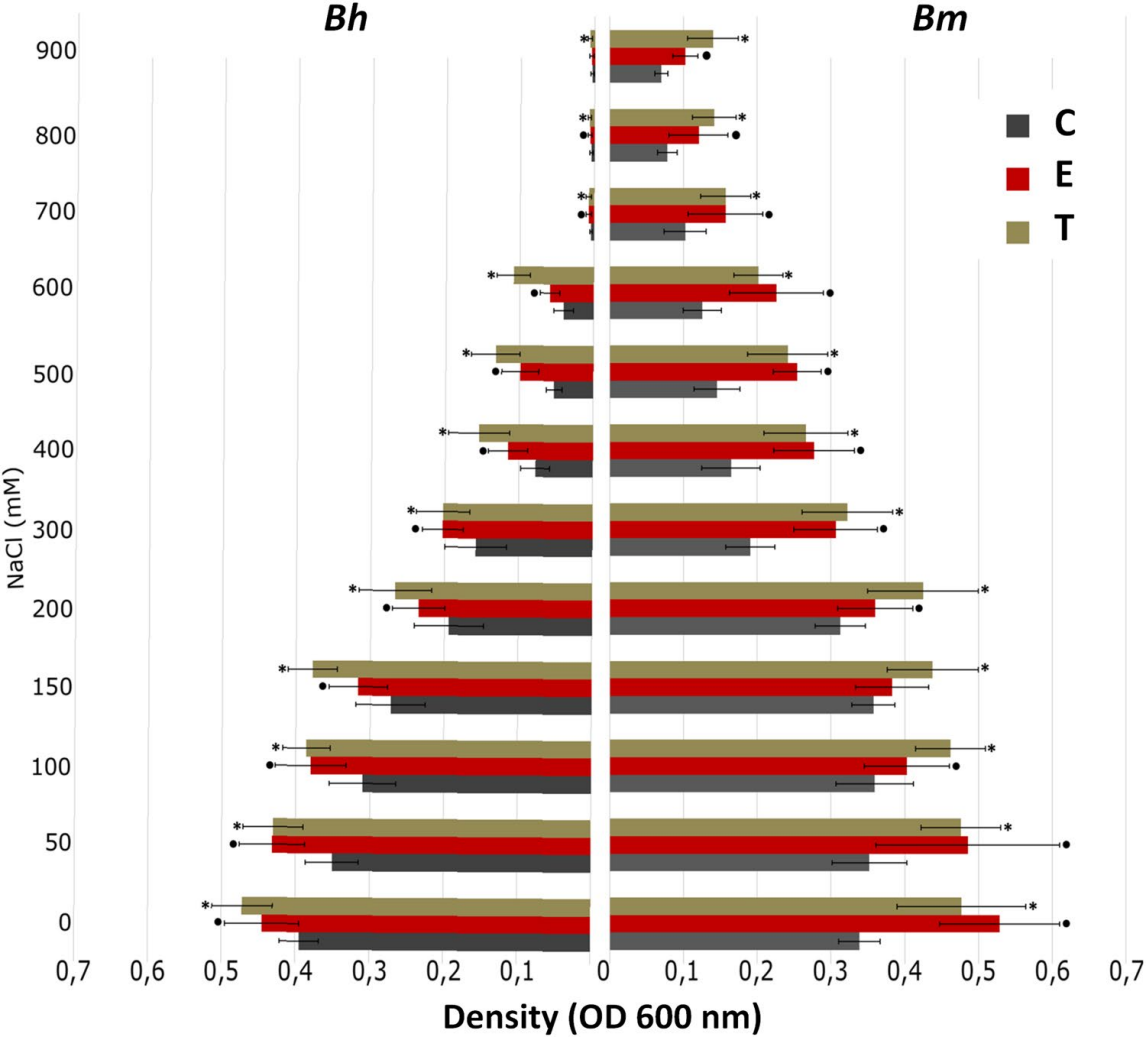
Osmolytes effect on growth of LB cultures inoculated with lyophilized sugar and sea beet roots untreated with any osmolyte (C), treated with ectoine (E) and trehalose (T). Means (n = 4–6) ± standard deviation are presented. Significant differences between treatments (ANOVA, p < 0.05, with Tukey's HSD) are marked with asterisks.

Increasing salinity negatively affected growth of culturable fraction of microbiome regardless of origin (sea- vs. sugar beet), however stronger effect was observed for sugar beet. In control treatment the growth was inhibited (final cell density below the critical level of 0.2 OD_600_) at 200 mM and 300 mM NaCl concentration for sugar and sea beet, respectively. Addition of osmolytes enhanced the growth in general and increased the inhibitory concentration to 400 and 700 mM, respectively (Supplementary Table 1). Influence of both osmolytes was similar, with trehalose performing slightly better at high NaCl concentrations., The effect was greater for sea beet, than for sugar beet (Fig. 4).

#### Bacterial viable cell density in lyophilized roots is associated with plant genotype and osmolyte

**Figure 5:**
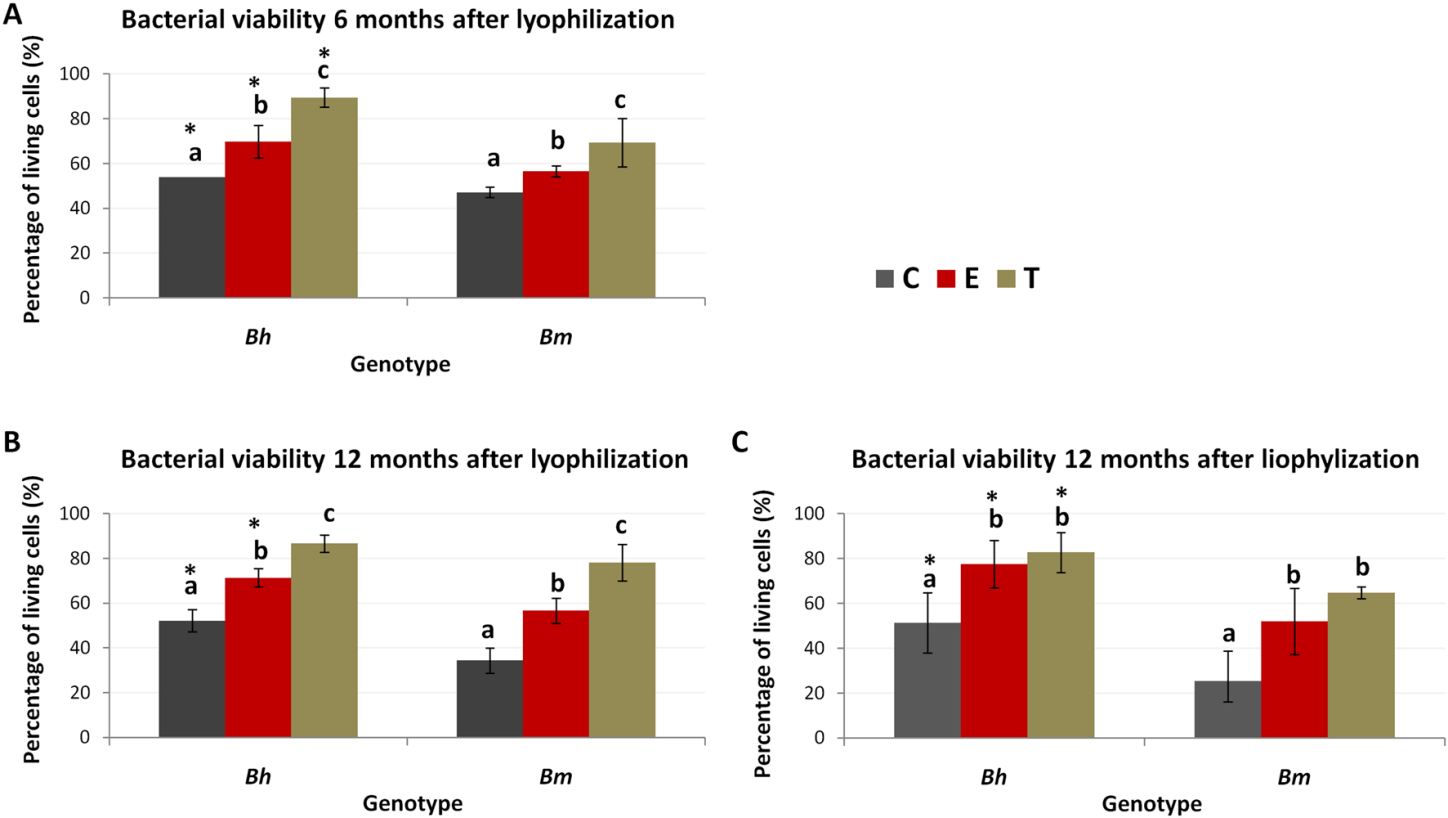
Bacterial viability in lyophilized beet roots. Viability measured with BD Cell Viability kit under fluorescence microscope (AB) and using flow cytometer (C). Means are presented and statistically significant differences between treatments are marked with differing letters. Stars denote significant differences between genotypes.

Cell viability in lyophilized beet roots was assessed by means of three, complementary methods: via plate counts, fluorescence microscopy and flow cytometry (Fig. 5). Bacterial viability in sugar beet was consistently higher than in roots of its wild relative, regardless of osmolyte treatment, storage time and measurement methodology. Both trehalose and ectoine increased the viability compared to control, regardless of genotype, but the effect of the former was more pronounced (Fig. 5).

## Discussion

### Bacterial diversity in beet rhizosphere

Differences in rhizosphere soil physicochemical properties observed in our study, may be due to greater nutritional demands of the two beet genotypes (TN, Na) or varying exudates composition (OC), as it was found that rhizodeposition is the primary organic carbon source in the rhizosphere^30^. Alternatively, they might be caused by changes in microbial activity resulting from microbial metabolic activity or interaction between microorganisms^31,32^.

Greater microbiome diversity in rhizosphere compared to endosphere was commonly observed, and resulted from natural plant selection mechanisms^33–35^. Accordingly, in our study, the higher bacterial diversity, evenness and species richness were noted in rhizosphere soil of both investigated genotypes, than in roots. At the same time, in spite of slightly different TN, OC and Na levels, microbiome composition and diversity were similar in rhizosphere soils of both studied plant genotypes. This observation could be explained by the use of the same starting substrate (garden soil) and short culture period (three months), not allowing the rhizosphere differences to fully manifest. Culture-independent analysis revealed that dominating bacterial phyla were the same as those observed in rhizosphere of many plant species e.g. barley, alfalfa or wheat^36–38^. Only a few differences between the genotypes were noted at the genus level, mainly concerning Alphaproteobacteria. *Pedomicrobium* as well as JG34.KF.361_ge, more frequent in sugar beet, represent *Rhizobiales,* an order known for organisms that establish beneficial interactions with plants and comprises numerous bacteria with nitrogen-fixing capability^39^. The observed lower TN level in the sugar beet rhizosphere may indicate higher demand for nitrogen. Tsurumaru and colleagues^40^ indicated that *Mesorhizobium* and *Bradyrhizobium,* also belonging to *Rhizobiales,* play an important ecological role in the taproot of sugar beet. Moreover, it was showed that higher levels of nitrogen (N) and potassium (K) significantly affect the growth parameters of sugar beet. Both elements were generally recognized as crucial for obtaining higher yields of this crop, favorably affecting organic metabolites biosynthesis and improving nutritional status^41^.

### Bacterial diversity in beet roots

The higher diversity both in rhizo- and endosphere of the wild plant compared to its crop counterpart was observed^42,43^. It was hypothesized that beneficial endophytes associated with wild plants were absent or fewer in domesticated crops^43^. Sugar beet as a cultivated plant grows under more controlled conditions regulated by farmers, while sea beet grows mainly in highly saline and nutrients poor coastal soil^28^. Growth under adverse environmental conditions requires support of microorganisms with a wide range of beneficial metabolic properties tailored for specific plant needs^23^. The loss of high tolerance to salt stress during the process of sea beet domestication was demonstrated^29^ and might be associated with the loss of microbes that increased tolerance of this plant to salinity. Concordantly, despite the lack of differences in rhizosphere soil microbial composition, lower diversity of endophytes in sugar beet compared to its wild ancestor was noted in our study. This difference might be explained by varying root system architecture, with fibrous root system of sea beet providing more opportunities for bacteria to enter the endosphere^33^, which affects stochastic community assembly. On the other hand microbe selection can be driven by the genetic makeup of two studied subspecies. We observed that sea beet caused decrease in the soil Na level, suggesting accumulation of Na ions in wild plant tissues. Accordingly, there was an increase in community salinity resistance in this plant, which pointed at higher level of halotolerant and halophytic microorganisms.

In general, endophytic microbiome diversity and composition is related to soil properties as well as plant ecology and physiology^44^. Members of only three phyla (Proteobacteria, Actinobacteria and Firmicutes) were cultured in our experiment, this may be related to their high ability to grow on commercially available media^5,6,44,45^. It was emphasized that Proteobacteria distinctly predominate among culturable plant endophytes, then the presence of Firmicutes and Actinobacteria is common, and Bacteroidetes occur slightly less frequently^44^.

16S rRNA gene libraries generated in our study were dominated by the four phyla (Proteobacteria, Actinobacteria, Firmicutes, Bacteroidetes) commonly found in endosphere of glycophytes including maize (*Zea mays* L.^46^), *Dactylis glomerata* L., *Festuca rubra* L. and *Lolium perenne* L.^47^ as well as in halophytes such as *Salicornia europaea*^23^ or para grass (*Urochloa mutica*^48^). Sea beet was characterized by significantly higher frequency of Actinobacteria, Bacteroidetes, Acidobacteria, Verrucomicrobia and rare phyla compared to sugar beet, where Proteobacteria were observed more often. Zachow et al. observed greater frequency of Actinobacteria, Bacteroidetes and Verrucomicrobia in rhizosphere of wild beet cultivated in coastal soil than in sugar beet rhizosphere^42^. This fact, together with our results, may point at these bacterial taxa being preferred by sea beet regardless of soil.

Our 16S rRNA gene sequencing results also revealed significantly higher abundance of certain genera in sea beet endosphere, including: *Novosphingobium*, *Devosia* (Alphaproteobacteria), *Hydrogenophaga*, *Polaromonas* (Betaproteobacteria), *Rhizobacter* and *Tahibacter* (Gammaproteobacteria) as well as certain rare and unclassified bacteria. This set of microorganisms comprises extremophiles, e.g. *Polaromonas*^49^ or *Hydrogenophaga*^50^ and organisms modulating plant stress response, such as *Novosphingobium*^25^. In our study, only *Stenotrophomonas* and *Bacillus* genera were more frequent in roots of sugar beet than of sea beet. *Stenotrophomonas* and *Pseudomonas* sp. were identified in rhizospheric soil of sugar and sea beet, while the former together with *Staphylococcus* sp. were mainly observed in crop rhizosphere. Sea beet microbiome was found to be more diverse than that of sugar beet, which is explained by greater number of rare taxa. It was found that sugar beet rhizosphere was more frequently colonized by strains with antagonistic activity against plant pathogens and/or stress protection activity, while abiotic stress-releasing ones were more often found in sea beet’s rhizosphere^42^. These facts together with our results suggest that pre-adaptation to stress observed in sea beet transcriptome^51^ may also take place at the level of microbiome serving as a helper.

### Osmoprotectants enhance bacterial viability and diversity in lyophilized beet roots

Significantly higher cell density of culturable bacteria observed in sugar beet lyophilized roots can be attributed to high content of sucrose. This sugar acts as a natural osmoprotectant, allowing better viability of microorganisms during lyophilization^52^. Another explanation of obtained results can be associated with higher ability of sugar beet endophytes to grow on solid medium.

Sea beet endophytic microbiome was found to be more resistant to salinity. Microorganisms present in a more saline sea beet tissue most likely developed mechanisms of adaptation to high salt level, which provided them ability to grow in higher NaCl concentrations compared to the sugar beet microbiome. This fact may be related to higher sodium accumulation in this plant tissues^51^, which caused decrease of soil sodium concentration observed in our study.

Salinity-induced changes in community structure and adverse effects on microbial density, activity, biomass were reported by many scientists^53,54^. The decrease in number of culturable microorganisms related to increasing NaCl concentration was noted even in the case of endophytes associated with halophytes (*Aster tripolium*, *Salicornia europaea*)^5,6,55^. Obtained results were in line with the above trend, but apart from negative effect of salinity on sugar and sea beet bacterial density, a beneficial impact of trehalose and ectoine on salt stress mitigation was demonstrated. Although ectoine is a major osmolyte in aerobic chemoheterotrophic bacteria and is considered as a marker for halophytic bacteria^15^, a slightly better effect of trehalose, was confirmed by the results of microscopic analyzes, flow cytometry and culture tests. Protective effect of trehalose is explained by “water replacement hypothesis” that states that the compound lowers the phase transition temperature of membrane phospholipids, by replacement of water molecules occurring around the lipid head groups^56^, thus protecting membrane structure^57^. This suggests that the use of trehalose is a better and more economic solution providing high viability of bacterial cells after lyophilization. In the case of sugar beet the above mentioned positive sucrose impact was enhanced by trehalose addition. Similar effect was observed for rhizobial strains, where trehalose worked better than sucrose/peptone mixture^58^. In general, 16S rRNA gene sequencing results considering diversity of endophytes associated with sea and sugar beet root did not show any effect of applied osmoprotectants neither on alpha nor beta diversity of bacteria. This observation can be explained by the presence of ‘relic DNA’, i.e. DNA coming from non-viable cells^59^ in lyophilized samples.

*Bacillus* sp. was the only species identified among the strains representing the Firmicutes phylum isolated from the lyophilized osmolytes-treated roots of both investigated genotypes. In the control variant the presence of *Psychrobacillus* sp. and *Paenibacillus* sp. inside sea and sugar beet root was additionally found, respectively. The viability of the above-mentioned bacteria after lyophilization was probably associated with their commonly known ability to form endospores and higher tolerance to environmental changes^60–62^. Actinobacteria proved to be sensitive to lyophilization, while Proteobacteria remarkably well tolerated it, and additional osmolytes promoted the incidence of culturable bacteria belonging to the latter phylum.

## Conclusions

Our research revealed that plant genotype played a pivotal role in the shaping of its endophytic microbiome diversity and physicochemical rhizosphere soil properties, affecting soil sodium content, but not soil bacterial community structure. Bacterial diversity was lower in sugar beet roots than in its wild ancestor tissues. At the same time sea beet endophytic microbiome was more salt resistant and consisted of genera characteristic for extreme environments.

Supplementing osmoprotectants during root tissue lyophilization had a positive effect on bacterial salt stress tolerance, viability and density. Trehalose proved to improve these parameters more effectively than ectoine, moreover its use was economically advantageous.

## Materials and methods

### Experimental design

**Figure 6:**
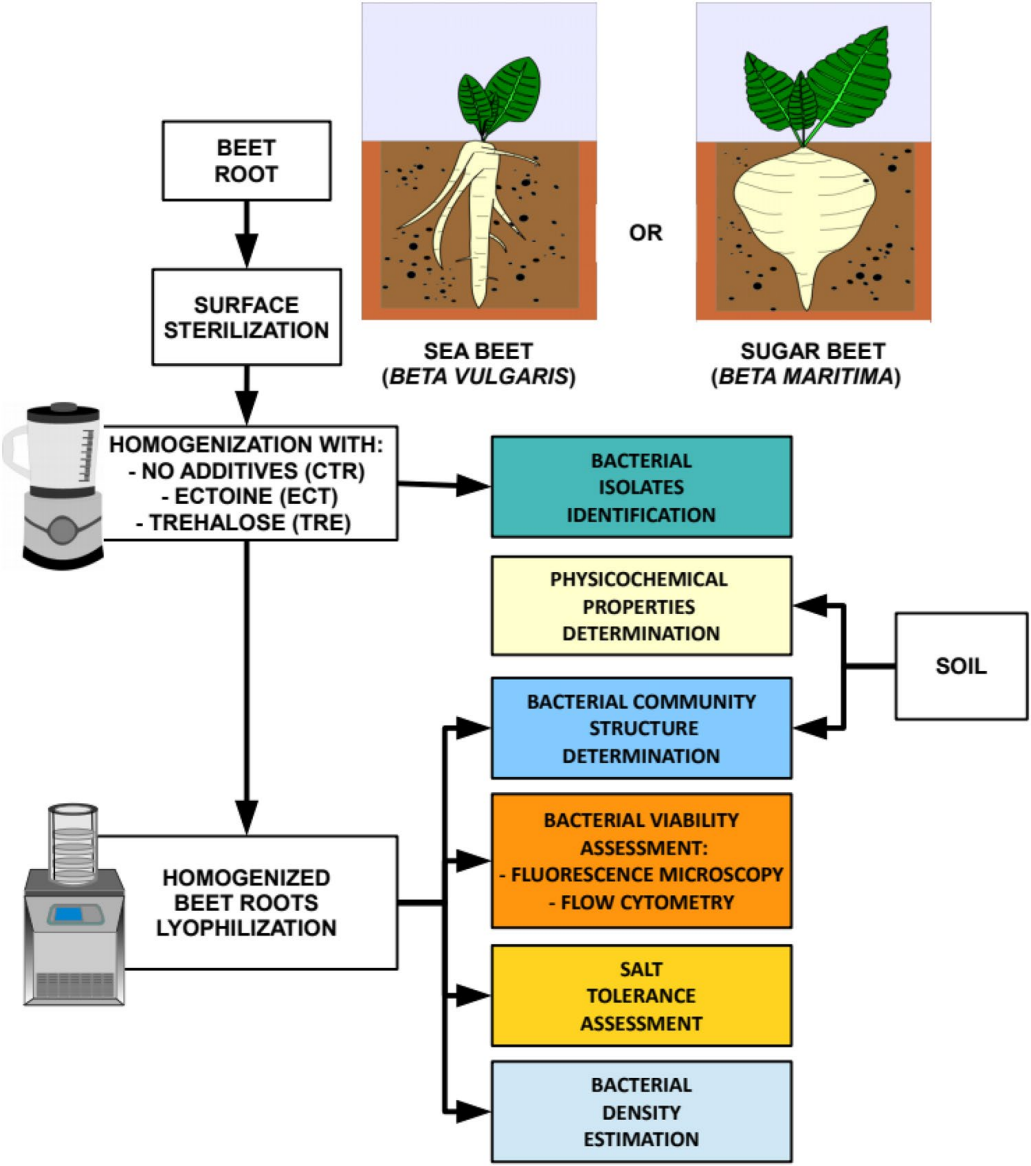
Experimental design.

Sea beet (*Beta vulgaris* L. subsp. *maritima* L.) seeds were obtained from National Germplasm Resources Laboratory, Beltsville, MD, USA**,** while in the case of sugar beet (*B. vulgaris* subsp. *vulgaris* cv. 'Huzar') commercial seeds were bought from WHBC Poznań, Poland. Healthy and uniform-sized seeds were placed in 5 l pots filled with 2.5 kg of garden soil. From twenty plants, five representative ones (with two pairs of true leaves and similar in size) were chosen for analysis. Pot experiment was conducted from mid-March through mid-May 2017 in a greenhouse (Nicolaus Copernicus University in Toruń, Poland). Plants were grown under natural lighting conditions and temperature was maintained at 22–24 °C throughout the growth period. All plants were arranged randomly on the green house benches. The plants were watered with tap water every two days, amount depended on the plants demand. After three months plants and rhizosphere soil samples were collected and analyzed as shown in Fig. 6.

### Soil analysis

Soil parameters (TOC, TN, CaCO_3_, P_citr_, pH, EC, Na, K, Ca, Mg, Cl, SO_4_^2−^) were analyzed as described earlier in Furtado et al.^27^.

### Plant and soil samples preparation

Plants were carefully uprooted, and 10 g of soil adhering to roots (rhizospheric soil) was collected, frozen at − 80 °C and lyophilized before DNA isolation for metagenomic analysis. Roots were washed with tap water to remove soil and were separated from shoots and leaves. Then, they were surface sterilized with 70% ethanol and 15% hydrogen peroxide mixture (1:1 v:v) for 5 min and subsequently rinsed six times with 0.9% NaCl. Efficiency of the sterilization process was evaluated by plating the last rinse on Luria*–*Bertani (Difco LB Agar, Miller) and potato dextrose extract (Lab A Neogen Company) media. Only properly sterilized plant material was used for subsequent analyzes. Approximately 100 g of fresh root material was homogenized in 100 ml of 0.9% NaCl by using surface sterilized (rinsed with 70% ethanol and UV-irradiated) blender. Homogenates were used to evaluate bacterial density and to prepare lyophilizates.

### Roots lyophilization

Homogenized sugar and sea beet roots were used to prepare three variants of lyophilizates including (1) no osmolytes addition (control—C) (2) trehalose (T) and (3) ectoine (E) supplemented. Three biological replicates were prepared for each tested plant species (9 samples per plant species, in total 18 samples were used for downstream analyzes). Either 1 ml of 0.9% NaCl (control) or 1.0 mg of trehalose (Tre) or 1.0 mg of ectoine (Ect) were mixed with 50 g of homogenized roots. The mixtures were lyophilized in Telstar LyuQues (DanLab) until completely dry (approximately 24 h).

### Estimation of bacterial density

Serial dilutions were prepared directly from the homogenized fresh roots and lyophilizates re-suspended in 0.9% NaCl (1:9 m:v). The dilutions (10^−3^ to 10^−8^) were plated in triplicates on LB plates supplemented with nystatin (Sigma, 100 µg/ml) to prevent fungal growth, and the plates were incubated for 5 days at 26 °C. Colony counts (expressed as CFU per 1 g of fresh or dry weight for homogenates and lyophilizates, respectively) were based on plates with 30–300 colonies. At least six bacterial isolates were purified per experimental variant.

### Bacterial viability assessment: fluorescence microscopy and flow cytometry

Ten-miligram samples of ground lyophilized roots were mixed with 10 ml of PBS (pH = 7.4) and incubated for 2 days at 26 °C with mixing. The mixtures were filtered through a 40 µm cell strainer (Biologix) and 2 ml were centrifuged for 3 min at 1000 × g at RT to pellet the residual plant debris. Cells in the supernatant were stained with Cell Viability kit (BectonDickinson) as per the manufacturer's protocol, than bacterial viability was analyzed using fluorescence microscopy (after 6 and 12 months of storage) and flow cytometer (after 12 months storage). Preparations were photographed in red and green channel under 40 × magnification upon fluorescence excitation with 433 nm light on Axiostar plus fluorescence microscope (Zeiss) equipped with Delta Optical camera. Percentage of live cells was based on counts from at least 30 view fields per sample. Flow cytometric analysis was performed on samples stained as described above with FACS Aria III (BectonDickinson) using 488 nm laser for excitation. Fluorescence was collected at 530 ± 30 nm (for thiazole orange—TO) and 616 ± 26 nm (for propidium iodide—PI) bands and seventy-micrometer nozzle was used. Parameters were optimized basing on pure environmental strains and their mixtures analyses and autoclaved lyophilizate samples served as negative controls.

### Salt tolerance assessment

Salt tolerance of root bacterial communities was measured as OD_600_ after 5 days incubation at 26 °C using 96-wells microtiter plate reader (Biolog Micro Station). 140 µl of LB medium supplemented NaCl to obtain final concentrations of 0, 50, 100, 150, 200, 300, 400, 500, 600, 700, 800, 900 mM were used per well. Inoculates were prepared by suspending 2 g of mortar-ground lyophilized roots in 18 ml of 0.9% NaCl and diluting the mixture ten times. The inoculates were filtered through 40 µm cell strainer (Biologix) to remove plant debris. Six test and two control wells were inoculated with 10 µl of filtered inoculate or 0.9% NaCl, respectively.

### Isolates identification by 16S rRNA gene sequencing

Genomic DNA was isolated from purified strains using GeneMatrix Bacterial and Yeast Genomic DNA Purification Kit (EurX) according to the manufacturer's protocol with modified homogenization step (FastPrep-24 bead-beater, one cycle of 20 s at 4.0 m/s). The DNA was analyzed spectrophotometrically (NanoDrop 2000). 16S rRNA gene fragment was amplified using 27F and 1492R primers^63^, following the procedure described in Szymańska et al.^6^. The products were purified with GeneMatrix PCR/DNA Clean-Up DNA Purification Kit (EurX) according to the manufacturer’s protocol. Sanger sequencing was performed with BrightDye Cycle Sequencing kit (Nimagen), using 40 ng of template DNA, 1.5 pmol of primer and 1 µl of kit and 1.5 µl of BD buffer in 10 µl volume. The reactions were EtOH/NaAc precipitated and read out at IBB PAS, Warsaw, Poland.

### 16S rRNA gene fragment library construction and sequencing

Metagenomic DNA was isolated and V3-V4 16S rRNA gene fragment libraries for Illumina sequencing were prepared as described earlier^64^. They were sequenced on Illumina MiSeq using 600 cycles v.3 kit at CMIT NCU.

### Statistical analysis and bioinformatics

Bioinformatics analyses of Illumina reads was performed as described earlier^64^. Briefly, the reads were denoised, merged and chimeras were removed with dada2^65^, then amplicon variant sequences were exported together with abundance information and processed in Mothur v.1.39^66^: aligned against SILVA v.132 database, screened for those covering the 6428-22400 positions of the alignment, filtered to remove gap-only and terminal gap-containing positions, pre-clustered to remove residual noise and clustered into 0.03 dissimilarity OTUs. Representative OTU sequences were classified using naïve Bayesian classifier^67^ and SILVA database^68^. Sanger reads were manually inspected in Chromas to remove obvious errors, the corrected sequences were merged with CAP3^69^, and classified using naïve Bayesian classifier with SILVA v.132 reference files.

Significance of differences between means was assessed with ANOVA test with Tukey's post-hoc analysis implemented in Statistica 10.0 (StatSoft). Normality of data was tested with Shapiro–Wilk's test and homogeneity of variance was assessed with Levene's test. When the assumptions were violated, non-parametric Kruskal–Wallis test with Dunn’s test as a post-hoc analysis was used. Significance level of 0.05 was assumed.

## Supplementary Information

Supplementary Information.

## Data Availability

Sequences generated during this study were deposited in the SRA repository and are accessible via BioProject no. PRJNA606174.

## References

[1] Gveroska B, Miceska G, Dimitrieski M, Korubin-Aleksoska A (2014). Use of biopreparates in Tobacco protection: contribution to sustainable agriculture. Türk Tarım ve Doğa Bilim. Derg..

[2] Baez-Rogelio A, Morales-García YE, Quintero-Hernández V, Muñoz-Rojas J (2017). Next generation of microbial inoculants for agriculture and bioremediation. Microb. Biotechnol..

[3] Pretty J (2008). Agricultural sustainability: concepts, principles and evidence. Philos. Trans. R. Soc. Lond. B. Biol. Sci..

[4] Malusá E, Sas-Paszt L, Ciesielska J (2012). Technologies for Beneficial Microorganisms Inocula Used as Biofertilizers. Sci. World J..

[5] Szymańska S (2016). Metabolic potential and community structure of endophytic and rhizosphere bacteria associated with the roots of the halophyte *Aster tripolium* L. Microbiol. Res..

[6] Szymańska, S., Płociniczak, T., Piotrowska-Seget, Z. & Hrynkiewicz, K. Endophytic and rhizosphere bacteria associated with the roots of the halophyte *Salicornia europaea* L. – community structure and metabolic potential. *Microbiol. Res.* **192**, 37–51 (2016). 10.1016/j.micres.2016.05.012 27664722

[7] Hrynkiewicz, K. & Patz, S. *Salicornia europaea* L. as an underutilized saline-tolerant plant inhabited by endophytic diazotrophs. *J. Adv. Res.* **19**, 49–56 (2019). 10.1016/j.jare.2019.05.002 PMC6630021 31341669

[8] Alori ET, Babalola OO (2018). Microbial inoculants for improving crop quality and human health in Africa. Front. Microbiol..

[9] Prakash O, Nimonkar Y, Shouche YS (2013). Practice and prospects of microbial preservation. FEMS Microbiol. Lett..

[10] Park JE, Lee KH, Jahng D (2002). Effect of trehalose on bioluminescence and viability of freeze-dried bacterial cells. J. Microbiol. Biotechnol..

[11] Reina-Bueno M (2012). Role of trehalose in salinity and temperature tolerance in the model halophilic bacterium *Chromohalobacter salexigens*. PLoS ONE.

[12] Lee H-J, Yoon Y-S, Lee S-J (2018). Mechanism of neuroprotection by trehalose: controversy surrounding autophagy induction. Cell Death Dis..

[13] Oren A (2008). Microbial life at high salt concentrations: phylogenetic and metabolic diversity. Saline Systems.

[14] Han J (2018). Transcriptomic and ectoine analysis of halotolerant *Nocardiopsis gilva* YIM 90087T under salt stress. Front. Microbiol..

[15] Roberts MF (2005). Organic compatible solutes of halotolerant and halophilic microorganisms. Saline Systems.

[16] Czech, L. *et al.* Role of the extremolytes ectoine and hydroxyectoine as stress protectants and nutrients: genetics, phylogenomics, biochemistry, and structural analysis. *Genes (Basel).* **9**, 177 (2018). 10.3390/genes9040177 PMC5924519 29565833

[17] Parnell JJ (2016). From the lab to the farm: an industrial perspective of plant beneficial microorganisms. Front. Plant Sci..

[18] Compant S, Samad A, Faist H, Sessitsch A (2019). A review on the plant microbiome: ecology, functions, and emerging trends in microbial application. J. Adv. Res..

[19] Hardoim, P. R. & van Elsas, J. D. Properties of Bacterial Endophytes Leading to Maximized Host Fitness. in *Molecular Microbial Ecology of the Rhizosphere* 405–411 (John Wiley & Sons, Inc., 2013). doi:10.1002/9781118297674.ch37

[20] Patle P (2018). Endophytes in plant system: Roles in growth promotion, mechanism and their potentiality in achieving agriculture sustainability. Int. J. Chem. Stud..

[21] Bencherif K (2015). Impact of soil salinity on arbuscular mycorrhizal fungi biodiversity and microflora biomass associated with *Tamarix articulata* Vahll rhizosphere in arid and semi-arid Algerian areas. Sci. Total Environ..

[22] Abbas, H., Patel, R. M. & Parekh, V. R. Culturable endophytic bacteria from halotolerant *Salvadora persica* L.: isolation and plant growth promoting traits. *Indian J. Appl. Microbiol.* **10**, 1074 (2018).

[23] Szymańska S (2018). Bacterial microbiome of root-associated endophytes of *Salicornia europaea* in correspondence to different levels of salinity. Environ. Sci. Pollut. Res..

[24] Yadav AN, Saxena AK (2018). Biodiversity and biotechnological applications of halophilic microbes for sustainable agriculture. J. Appl. Biol. Biotechnol..

[25] Etesami H, Beattie GA (2018). Mining halophytes for plant growth-promoting halotolerant bacteria to enhance the salinity tolerance of non-halophytic crops. Front. Microbiol..

[26] Szymańska, S. *et al.* Boosting the *Brassica napus* L. tolerance to salinity by the halotolerant strain *Pseudomonas stutzeri* ISE12. *Environ. Exp. Bot.* **163**, 55–68 (2019).

[27] Furtado, B. U., Gołębiewski, M., Skorupa, M., Hulisz, P. & Hrynkiewicz, K. Bacterial and fungal endophytic microbiomes of *Salicornia europaea*. *Appl. Environ. Microbiol.* **85**, (2019). 10.1128/AEM.00305-19 PMC6581177 31003988

[28] Dohm JC (2014). The genome of the recently domesticated crop plant sugar beet (*Beta vulgaris*). Nature.

[29] Rozema, J. *et al.* Comparing salt tolerance of beet cultivars and their halophytic ancestor: consequences of domestication and breeding programmes. *AoB Plants* **7**, (2014). 10.1093/aobpla/plu083 PMC4381740 25492122

[30] Bashir, O. *et al.* Soil microbe diversity and root exudates as important aspects of rhizosphere ecosystem. in *Plant, Soil and Microbes* 337–357 (Springer International Publishing, 2016). 10.1007/978-3-319-29573-2_15

[31] Nannipieri, P. *et al.* Effects of root exudates in microbial diversity and activity in rhizosphere soils. in 339–365 (Springer, Berlin, Heidelberg, 2008). 10.1007/978-3-540-75575-3_14

[32] Shi S (2011). Effects of selected root exudate components on soil bacterial communities. FEMS Microbiol. Ecol..

[33] Kandel S, Joubert P, Doty S (2017). Bacterial endophyte colonization and distribution within plants. Microorganisms.

[34] Liu H (2017). Inner plant values: diversity, colonization and benefits from endophytic bacteria. Front. Microbiol..

[35] Cheng, D., Tian, Z., Feng, L., Xu, L. & Wang, H. Diversity analysis of the rhizospheric and endophytic bacterial communities of *Senecio vulgaris* L. (Asteraceae) in an invasive range. *PeerJ* **6**, e6162 (2019). 10.7717/peerj.6162 PMC6327885 30643678

[36] Velázquez-Sepúlveda, I., Orozco-Mosqueda, M. C., Prieto-Barajas, C. M. & Santoyo, G. Bacterial diversity associated with the rhizosphere of wheat plants (*Triticum aestivum*): Toward a metagenomic analysis. *Phyton (B. Aires).* **81**, 81–87 (2012).

[37] Bulgarelli D (2015). Structure and function of the bacterial root microbiota in wild and domesticated barley. Cell Host Microbe.

[38] Kumar V (2018). Metagenomic analysis of rhizosphere microflora of oil-contaminated soil planted with barley and alfalfa. PLoS ONE.

[39] Erlacher A (2015). Rhizobiales as functional and endosymbiontic members in the lichen symbiosis of *Lobaria pulmonaria* L. Front. Microbiol..

[40] Tsurumaru H (2015). Metagenomic analysis of the bacterial community associated with the taproot of sugar beet. Microbes Environ..

[41] Abdel-Motagally FMF, Attia KK (2009). Response of sugar beet plants to nitrogen and potassium fertilization in sandy calcareous soil. Int. J. Agric. Biol..

[42] Zachow, C., Mueller, H., Tilcher, R. & Berg, G. Differences between the rhizosphere microbiome of *Beta vulgaris* ssp. *maritima*-ancestor of all beet crops-and modern sugar beets. *Front. Microbiol.* **5**, 415 (2014). 10.3389/fmicb.2014.00415 PMC4144093 25206350

[43] Ofek-Lalzar, M. *et al.* Diversity of fungal endophytes in recent and ancient wheat ancestors *Triticum dicoccoides* and *Aegilops sharonensis*. *FEMS Microbiol. Ecol.* **92**, fiw152 (2016). 10.1093/femsec/fiw152 27402714

[44] Miliute I, Buzaite O, Baniulis D, Stanys V (2015). Bacterial endophytes in agricultural crops and their role in stress tolerance: a review. Zemdirbyste Agricu..

[45] Brígido, C. *et al.* Diversity and Functionality of Culturable Endophytic Bacterial Communities in Chickpea Plants. *Plants (Basel, Switzerland)* **8**, (2019). 10.3390/plants8020042 PMC6409739 30769814

[46] Correa-Galeote D, Bedmar EJ, Arone GJ (2018). Maize endophytic bacterial diversity as affected by soil cultivation history. Front. Microbiol..

[47] Wemheuer F (2017). Bacterial endophyte communities of three agricultural important grass species differ in their response towards management regimes. Sci. Rep..

[48] Mukhtar S (2016). Microbial diversity and metagenomic analysis of the rhizosphere of para grass (*Urochloa mutica*) growing under saline conditions. Pakistan J. Bot..

[49] Gawor J (2016). Evidence of adaptation, niche separation and microevolution within the genus *Polaromonas* on Arctic and Antarctic glacial surfaces. Extremophiles.

[50] Khan, M. & Goel, R. Principles, applications and future aspects of cold-adapted PGPR. in *Plant-Bacteria Interactions* 195–212 (Wiley-VCH Verlag GmbH & Co. KGaA, 2008). 10.1002/9783527621989.ch10

[51] Skorupa, M. *et al.* Salt stress vs. salt shock-the case of sugar beet and its halophytic ancestor. *BMC Plant Biol.* 1–18 (2019). 10.1186/s12870-019-1661-x PMC6364445 30727960

[52] Bircher L, Geirnaert A, Hammes F, Lacroix C, Schwab C (2018). Effect of cryopreservation and lyophilization on viability and growth of strict anaerobic human gut microbes. Microb. Biotechnol..

[53] Yan N, Marschner P, Cao W, Zuo C, Qin W (2015). Influence of salinity and water content on soil microorganisms. Int. Soil Water Conserv. Res..

[54] Zhang, K. *et al.* Salinity Is a key determinant for soil microbial communities in a desert ecosystem. *mSystems* **4**, (2019). 10.1128/mSystems.00225-18 PMC6372838 30801023

[55] Szymańska, S., Piernik, A. & Hrynkiewicz, K. Metabolic potential of microorganisms associated with the halophyte *Aster tripolium* L. in saline soils. *Ecol. Quest.* **18**, 9–19 (2013).

[56] Berninger, T., González López, Ó., Bejarano, A., Preininger, C. & Sessitsch, A. Maintenance and assessment of cell viability in formulation of non-sporulating bacterial inoculants. *Microb. Biotechnol.* **11**, 277–301 (2018). 10.1111/1751-7915.12880 PMC5812248 29205959

[57] Nounjan N, Theerakulpisut P (2012). Effects of Exogenous proline and trehalose on physiological responses in rice seedlings during salt-stress and after recovery. Plant, Soil Environ..

[58] Arraes Pereira, P. A., Oliver, A., Bliss, F. A., Crowe, L. & Crowe, J. Preservation of rhizobia by lyophilization with trehalose. *Pesqui. Agropecu. Bras.* **37**, 831–839 (2002).

[59] Carini P (2017). Relic DNA is abundant in soil and obscures estimates of soil microbial diversity. Nat. Microbiol..

[60] Nicholson WL, Munakata N, Horneck G, Melosh HJ, Setlow P (2000). Resistance of *Bacillus* endospores to extreme terrestrial and extraterrestrial environments. Microbiol. Mol. Biol. Rev..

[61] Pham, V. H. T., Kim, J. & Jeong, S.-W. *Psychrobacillus soli* sp. nov., capable of degrading oil, isolated from oil-contaminated soil. *Int. J. Syst. Evol. Microbiol.* **65**, 3046–3052 (2015). 10.1099/ijs.0.000375 26065735

[62] Sáez-Nieto, J. A. *et al. Paenibacillus* spp. isolated from human and environmental samples in Spain: detection of 11 new species. *New Microbes New Infect.* **19**, 19–27 (2017). 10.1016/j.nmni.2017.05.006 PMC5484988 28702198

[63] Lane DJ, Stackebrandt E, Goodfellow M (1991). 16S/23S rRNA sequencing. Nucleic acid techniques in bacterial systematics.

[64] Thiem D, Gołębiewski M, Hulisz P, Piernik A, Hrynkiewicz K (2018). How does salinity shape bacterial and fungal microbiomes of *Alnus glutinosa* roots?. Front. Microbiol..

[65] Callahan BJ (2016). DADA2: high-resolution sample inference from Illumina amplicon data. Nat Methods..

[66] Schloss PD (2009). Introducing mothur: Open-source, platform-independent, community-supported software for describing and comparing microbial communities. Appl. Environ. Microbiol..

[67] Wang Q, Garrity GM, Tiedje JM, Cole JR (2007). Naive Bayesian Classifier for Rapid Assignment of rRNA Sequences into the New Bacterial Taxonomy. Appl. Environ. Microbiol..

[68] Quast C (2013). The SILVA ribosomal RNA gene database project: Improved data processing and web-based tools. Nucleic Acids Res..

[69] Huang X, Madan A (1999). CAP3: A DNA Sequence Assembly Program. Genome Res..

