# Supplementary material for: Choosing source of microorganisms and processing technology for next generation beet bioinoculant
Source: Sci Rep. 2021 Feb 2;11:2829. doi: 10.1038/s41598-021-82436-5 (PMC7854725; doi:10.1038/s41598-021-82436-5)
Supplement: Supplementary file 1 — Supplementary Information. [file 41598_2021_82436_MOESM1_ESM.docx]

**Choosing source of microorganisms and processing technology for next generation beet bioinoculant**

**Sonia Szymańska^1^, Marcin Sikora^2^, Katarzyna Hrynkiewicz^1*^, Jarosław Tyburski^2,3^, Andrzej Tretyn^2,3^, Marcin Gołębiewski^2,3*^**

1 – Department of Microbiology, Faculty of Biological and Veterinary Sciences, Nicolaus Copernicus University

2 – Center for Modern Interdisciplinary Technologies, Nicolaus Copernicus University

3 – Chair of Plant Physiology and Biotechnology, Faculty of Biological and Veterinary Sciences, Nicolaus Copernicus University

* - corresponding authors:

e-mail: [mgoleb@umk.pl](mailto:mgoleb@biol.umk.pl), Chair of Plant Physiology and Biotechnology NCU, Lwowska 1, 87-100 Toruń, Poland, phone: +48 56 611 2512, fax: +48 56 611 4559

e-mail: [hrynk@umk.pl](mailto:hrynk@umk.pl), Department of Microbiology, Faculty of Biology and Environmental Protection NCU, Lwowska 1, 87-100 Toruń, Poland, phone +48 611 25 40, fax: + 48 611 47 72

**Supplementary Table 1**. Optical density (at 600 nm) of LB cultures inoculated with beet roots lyophilized in presence of threhalose (T), ectoine (E) and with no additions (C). Means of eight replicates and SD (in parentheses) are given. Different letters denote statistically significant difference according to ANOVA and Tukey HSD (p<0.05).

|  | ***B. huzar*** | | | | ***B. maritima*** | | |
| --- | --- | --- | --- | --- | --- | --- | --- |
| NaCl (mM) | **C** | **E** | **T** | **C** | | **E** | **T** |
| 0 | 0,395  (0,0269) i | 0,445  (0,0502) i | 0,472  (0,0412) i | 0,338  (0,028) fg | | 0,528  (0,082) f | 0,477  (0,0875) f |
| 50 | 0,351  (0,0359) h | 0,432  (0,0443) h | 0,430  (0,0406) h | 0,352  (0,051) fg | | 0,485  (0,125) f | 0,476  (0,0537) f |
| 100 | 0,310  (0,0450) g | 0,380  (0,0482) g | 0,386  (0,0322) g | 0,359  (0,053) g | | 0,403  (0,058) e | 0,462  (0,0472) f |
| 150 | 0,272  (0,0472) f | 0,316  (0,0399) f | 0,378  (0,0332) f | 0,357  (0,029) g | | 0,383  (0,049) e | 0,438  (0,0616) f |
| 200 | 0,194  (0,0469) e | 0,235  (0,0355) e | 0,267  (0,0493) e | 0,312  (0,034) f | | 0,360  (0,051) de | 0,425  (0,0752) f |
| 300 | 0,158  (0,0418) d | 0,203  (0,0277) d | 0,202  (0,0359) d | 0,190  (0,033) e | | 0,306  (0,056) cd | 0,322  (0,0615) e |
| 400 | 0,078  (0,0195) c | 0,115  (0,0267) c | 0,154  (0,0415) c | 0,164  (0,040) de | | 0,277  (0,055) bc | 0,266  (0,0570) d |
| 500 | 0,053  (0,0106) bc | 0,099  (0,0250) c | 0,132  (0,0328) bc | 0,145  (0,031) cd | | 0,254  (0,032) bc | 0,241  (0,0542) Cd |
| 600 | 0,041  (0,0132) b | 0,059  (0,0135) b | 0,109  (0,0226) b | 0,125  (0,026) bc | | 0,226  (0,064) b | 0,201  (0,0333) bc |
| 700 | 0,004  (0,0015) a | 0,007  (0,0037) a | 0,007  (0,0039) a | 0,102  (0,028) ab | | 0,157  (0,051) a | 0,157  (0,0339) ab |
| 800 | 0,004  (0,0024) a | 0,006  (0,0025) a | 0,007  (0,0024) a | 0,078  (0,013) a | | 0,120  (0,040) a | 0,141  (0,0297) a |
| 900 | 0,003  (0,0021) a | 0,004  (0,0029) a | 0,007  (0,0029) a | 0,070  (0,009) a | | 0,102  (0,017) a | 0,140  (0,0346) a |
